# Supplementary material for: Optimization of transportation routing problem for fresh food in time-varying road network: Considering both food safety reliability and temperature control
Source: PLoS One. 2020 Jul 27;15(7):e0235950. doi: 10.1371/journal.pone.0235950 (PMC7384659; doi:10.1371/journal.pone.0235950)
Supplement: Attachment — Submitted filename: Manuscript_clean copy.docx [file pone.0235950.s001.docx]

**Research on TDVRPTW of Fresh Food Cold Chain Distribution Optimization: Considering both Food Safety Reliability and Temperature Control**

Zhixue ZHAO ^1,2*^, Xiamiao LI^1^, Xiancheng ZHOU ^2^

1. School of Traffic and Transportation Engineering, Central South University, Changsha Hunan, China

2. Key Laboratory of Hunan Province for Mobile Business Intelligence, Hunan University of Technology and Business, Changsha Hunan, China

**Abstract:** This study of fresh food safety reliability and temperature control is a research focus in the field of fresh food cold chain distribution optimization. On this basis, the time-dependent vehicle routing problem with time windows (TDVRPTW) of fresh food cold chain distribution is studied by considering both economic costs and fresh food safety losses. A calculation method for the path division strategy is designed. A food safety value loss measurement function, a metric function of energy and heat conversion, and a function of the carbon emission rate are employed by considering time-varying vehicle speeds, fuel consumption, the cost of temperature control, the loss of food safety reliability, and carbon emissions from transportation and temperature control. A TDVRPTW model is formulated based on the objective of minimizing the distribution cost and food safety value loss. According to the characteristics of the model, an adaptive improved ant colony algorithm is designed. Finally, the experimental data show that the model can effectively avoid the congestion period, reasonably control the refrigeration temperature, reduce the distribution cost, and improve food safety.

**Keywords:** cold chain distribution; time-varying; food safety value loss; path division strategy; temperature control; improved ant colony algorithm

**1. Introduction**

The new economic structure has stimulated the rapid development of the fresh food e-commerce industry. People have increasingly high standards for the quality of fresh food. The current fresh food logistics distribution system is still imperfect. The high logistics costs, the serious loss and highly reduced safety of fresh food, and the low transportation rate have become the key factors in cold chain logistics that constrain the development of fresh food e-commerce. As cities grow in size, traffic congestion is common. The optimal planning of cold chain logistics distribution routes can not only reduce distribution costs, ensure the hygiene and safety of fresh food, and increase customer satisfaction but also reduce energy consumption and carbon emissions, thus achieving environmental protection.

A critical issue in fresh food cold chain distribution is to scientifically solve the vehicle routing problem (VRP). With the advancement of VRP research, the fresh food cold chain distribution optimization problem has increasingly attracted the attention of researchers from around the world. Osvald et al. ^[1]^ introduced a function to measure the perishability of fresh vegetables and transform it to a cost. The researchers constructed a vehicle routing optimization problem model with time windows and verified its performance using Solomon’s instances. Chen ^[2]^ gave a full account of the perishability of fresh food by building a nonlinear programming model that considers production scheduling and distribution vehicle routing with time window constraints and solving the model with a designed heuristic algorithm. The optimal production quantities, the time to start producing, and the optimal distribution route were determined. Tiwari ^[3]^ optimized cold chain vehicle routes with the goal of minimizing travel distances and carbon emissions and solved the problem with an improved cluster algorithm. Tarantilis et al. ^[4]^ considered meat and milk distribution in Greece, studied a cold chain logistics distribution VRP with a heterogeneous fixed fleet of vehicles and multiple distribution centers, and used a threshold-accepting-based algorithm to solve the problem. Amorim ^[5]^ formulated a heterogeneous-fleet VRP with multiple time windows based on a food distribution problem in Portugal. Xiangguo ^[6]^ studied a vehicle routing optimization model of cold chain logistics based on stochastic demand. Lan et al. ^[7]^ analyzed the influence of urban traffic congestion on the costs of cold chain logistics vehicle distribution, constructed a corresponding vehicle routing optimization model, and designed a hybrid genetic algorithm to solve the model. Regarding uncertain demand, Li et al. ^[8]^ constructed a distribution routing optimization problem with time windows for perishable products based on stochastic customer demand. Ma ^[9]^ and Shao ^[10]^ solved the cold chain distribution VRP under fuzzy-stochastic demand with uncertain customer demands and time windows, and the routing optimization problem was solved for the overall distribution cost and customer satisfaction. Kang ^[11]^ built cold chain logistics transportation models that includes the costs of vehicle use, transportation costs, damage costs, refrigeration costs, and penalty costs and considers minimizing carbon emission costs as an optimization goal to optimize the distribution routing of cold chain logistics. Improved ant colony algorithms (IACAs), genetic algorithms, and other algorithms were used to solve the problem. Zhang ^[12]^ designed a cold chain logistics vehicle routing mathematical model with multiple depots and multiple types of vehicles and solved it with a genetic algorithm and elite selection. In a paper on perishable fresh product distribution, Ma et al. ^[13]^ constructed an integrated optimization model of production scheduling and distribution for perishable products to maximize profits and designed a hybrid intelligent algorithm to solve the model. Devapriya ^[14]^ formulated an integrated production and distribution scheduling optimization model for a perishable fresh product with the objective of minimizing the total logistics cost. A mixed heuristic algorithm was designed to resolve the model. Wu ^[15]^ studied the integrated production and distribution scheduling optimization problem with time windows of perishable food under time-varying network conditions. Wang et al. ^[16]^ focused on the deficiencies in a reasonable combination of the time windows of customers and the temperature control of fresh products in a study on fresh product logistics distribution optimization. Considering the stringent timeliness requirements of fresh product delivery, an optimization model was constructed to minimize both the distribution logistics cost and the value loss of fresh products. From the perspectives of both economic and environmental costs, Liu et al. ^[17]^ studied the VRP with time windows for fresh e-commerce distribution on time-varying networks. Their study comprehensively considered the time-varying velocity of vehicles, the perishability and spoilage of fresh agricultural products, and the time windows of customers and employed the ant colony algorithm (ACA) to solve the problem. Wang ^[18]^ investigated the optimization of the VRP with time windows for cold chain logistics based on a carbon tax in China and proposed a Cycle Evolutionary Genetic Algorithm (CEGA) to solve the model.

The current literature on food distribution systems mostly considers the physical losses generated in the process of food distribution due to perishability in the VRP. However, the physical loss of food and food safety are not the same concept. Although no physical changes in foods such as milk and sushi are evident, their safety could fail to meet sanitary standards. Zwietering ^[19]^ investigated a model of the influences of several temperatures on microbial growth in foods. Xie ^[20]^ introduced the reliability and safety of food cold chain logistics into the distribution routing optimization problem and solved the problem with a max-min ACA.

The results achieved so far have laid a good foundation for research on the cold chain logistics VRP. Nevertheless, limitations still exist in the following four main aspects. (1) The existing research results on cold chain logistics distribution generally assume a constant vehicle velocity, which is not consistent with real life. A few studies are based on time-varying networks. Additionally, most studies assume that vehicles leave the distribution center at the same time, while a few consider vehicles departing at different times. (2) Most of the existing research results on cold chain logistics distribution do not consider traffic congestion, which is common. Traffic congestion can significantly increase the driving time, vehicle energy consumption, carbon emissions, and loss of food safety, seriously affecting the distribution efficiency and city environment; thus, the study of congestion is urgent. (3) The current literature about cold chain logistics vehicle routing optimization generally only considers the physical loss of food due to perishability during fresh food distribution, while a few studies address the food safety issue, which ought to be considered in fresh food cold chain transportation distribution. (4) The current studies on fresh food logistics distribution mainly focus on optimization problems for time windows, fuel consumption, and customer satisfaction, while temperature-control-based problems associated with cold chain distribution, such as the temperature control cost and fresh food safety loss, need to be further studied.

This paper first analyzes the influence of time-varying networks on vehicle routing planning under traffic congestion. The velocity of refrigerated vehicles and the effect of loading on fuel consumption and carbon emissions are considered. A fresh food safety loss function is introduced. A temperature-control-based optimization model is constructed, with the objective of minimizing the logistics costs of fresh food distribution and fresh food safety losses. An ACA based on a route partitioning strategy is developed to solve the model. Through calculations and sensitivity analysis, the optimal distribution routes and optimal temperatures are determined, thus achieving highly efficient cold chain logistics and environmental protection.

**2. Problem description and model formulation**

## 2.1 Problem description and basic assumptions

Distribution centers, refrigerated transportation vehicles, and customer nodes constitute fresh food distribution networks. The problem is as follows. The distribution center distributes fresh food to customers. The locations, demands, and time windows of customer nodes are known. Distribution vehicles can arrive at customer nodes in advance but have to wait until the start of service time to serve the customers. There is one type of fresh food to distribute, and its safety constantly deteriorates. The traffic congestion periods and levels of the city can be obtained from the transportation department. The problem is to comprehensively consider the logistics distribution costs and determine the optimal vehicle dispatching and routing plan that minimizes the total distribution costs and maximizes the food safety. To facilitate the analysis, the following assumptions are made. (1) There is only one distribution center, which has sufficient supplies. There are many refrigerated transportation vehicles, which are of the same type. (2) Distribution vehicles depart from the cold chain logistics distribution center and return to the center immediately after completing the delivery task. (3) The demand of each customer node is less than the vehicle capacity. Service time windows are required. Vehicles cannot arrive later than the time window. They can arrive earlier but must wait until the start of the service window to distribute food. (4) During the periods of traffic congestion, the speed of vehicles is the typical speed under city traffic congestion conditions. During the no-congestion period, vehicles run at a normal speed. (5) The temperature inside the refrigerated vehicles is assumed to be stable and desirable during distribution. (6) The vehicle management costs include the vehicle running costs, labor costs, and vehicle usage costs. (7) The maximum load capacity of each vehicle is fixed. Each customer node has only one vehicle to serve it. While waiting to serve a customer, the engine is turned off to avoid fuel consumption and carbon emissions, but the temperature control equipment operates normally.

## 2.2 Solving for the driving time using the route partitioning strategy

Under a time-varying network with the driving speeds in different time periods given according to Ref. [21], we describe the time-dependent driving time model under time-varying traffic congestion conditions. The travel time of the vehicle on any road depends on changes in the driving speed related to the time of departure, as shown in Figure 1.

(a) (b)

Fig. 1. Continuous function of departure time based on driving speed

The planning horizon is divided into three consecutive periods: (1) a congestion period with a steady speed v_c_ (a constant), (2) a transition period, and (3) a free-flow period with a steady speed v_f_ (a variable). The travel time of arc (i, j), with distance d_ij_, congestion period (or free-flow period) start time a, and departing time w, is calculated as follows:

$t(w)=\left\{\begin{matrix} d/v_{c},if\text{ w}\leq\text{(a-}d/v_{c}\text{)}^{\text{+}} \\ \frac{v_{f}-v_{c}}{v_{f}}(a-w)+d/v_{f},\text{if(a-}d/v_{c}\text{)}^{\text{+}}\leq w\leq a\text{ } \\ d/v_{f},if\text{ w}\geq\text{a} \end{matrix}\right.$ (1)

$t(w)=\left\{\begin{matrix} d/v_{c},if\text{ w}\leq\text{(a-}d/v_{f}\text{)}^{\text{+}} \\ \frac{v_{f}-v_{c}}{v_{c}}(a-w)+d/v_{c},\text{if(a-}d/v_{f}\text{)}^{\text{+}}\leq w\leq a\text{ } \\ d/v_{f},if\text{ w}\geq\text{a} \end{matrix}\right.$ (2)

Formula (1) considers the situation Figure 1(a); Formula (2) considers the situation Figure 1(b). It is difficult to calculate the driving time.

According to Ref. [17,21,22], based on actual situations and driving patterns, if a vehicle runs on a sufficiently short section of a route, its speed on this section can be assumed to be constant. This paper analyzes the relationships between the driving time, vehicle velocity, route section, and driving distance based on the existing literature and designs a route partitioning method to solve the problem.

Step 1. Partition the route. First, the length of a sufficiently short route section is set to be $\vartheta$($\vartheta=0.2km$), and the vehicle speed on this route section is assumed to be constant. A vehicle k runs on route (i, j). The length of the route is $d_{ij}$. Route (i, j) is partitioned to a number of $\text{H}=\left\lceil d_{ij}\text{/}\vartheta\right\rceil$ route sections ( means rounding up to the nearest integer) according to $\vartheta$. The first H-1 route sections have a length of $\vartheta$, while the last section has a length of $d_{ij}\text{-}\vartheta\text{*（H-1）}$.

Step 2. Calculate the driving time on the first H-1 route sections. The time at which vehicle k leaves node i, $\text{T}_{_{\text{ik}}}^{leave}$, is used as the start time, $T_{_{\text{ijk1}}}^{leave}$, of driving into the first section of route (i, j). If $T_{_{\text{ijk1}}}^{leave}$ belongs to a congestion period, the vehicle speed for this route section is $v_{c}$, the driving time on this route section is $\text{t}_{_{\text{ijk1}}}^{d}=\vartheta/v_{c}$, and the time at which the vehicle arrives at this route section is used as the departure time from the second route section, $T_{_{\text{ijk2}}}^{leave}=T_{_{\text{ijk1}}}^{leave}+\vartheta/v_{c}$. Otherwise, the vehicle runs at a normal speed, with a driving time of $\text{t}_{_{\text{ijk1}}}^{d}=\vartheta/v_{f}$, and the departure time for the second route section is $T_{_{\text{ijk2}}}^{leave}=T_{_{\text{ijk1}}}^{leave}+\vartheta/v_{f}$. The driving times on the middle sections can be expressed in the same manner.

Step 3. Calculate the driving time on the last route section. If the departure time for the last route section, $T_{_{ijk\text{H}}}^{leave}$, belongs to a congestion period, the driving time is; otherwise, it is.

Step 4. Calculate the total driving time of vehicle k on route (i, j): $\text{t}_{_{ijk}}^{d}=\sum_{h=1}^{H} t_{_{ijkh}}^{d}$. The computation terminates.

## 2.3 Food safety loss measurement function

The primary issue in the process of cold chain distribution is the hygiene and safety of food. Once a food safety problem occurs during transportation, it could induce health events and cause panic. Foodborne diseases caused by pathogenic microorganisms have become the number one factor affecting food safety. Based on Ref. [20], the temperature inside the distribution vehicles is assumed to remain constant during the distribution process. The food safety loss, F_ijkw_, for vehicle k traveling from node i to node j at a controlled Celsius temperature w is

$F_{ijkw}\text{=d}\sum_{j\in L} q_{j}\cdot\Delta W^{2}t_{ijk},\text{ }t_{ijk}\text{=}t_{_{ijk}}^{\text{d}}\text{+}\eta_{jk}\text{+}t_{_{jk}}^{s}$ (3)

where d denotes the safety influence coefficient, the value of which is determined by the type of food, the cold chain logistics environment, and the type of pathogenic microorganism; qj is the demand of customer node j; $\Delta W=\text{w-W}_{\min}$, where w is the controlled temperature of the current refrigerated vehicle (Celsius), and W_min_ is the temperature at which no microorganisms grow (Celsius); and the distribution time, t_ijk_, includes the driving time $t_{_{ijk}}^{\text{d}}$ from node i to node j, the waiting time $\eta_{jk}$ at customer node j (if arriving in advance), and the unloading time $t_{_{jk}}^{s}$ needed to serve node j. Due to differences in customer demand, the total safety loss depends on the controlled temperature, the distribution time, and the customer service order.

Let n_k_ be the number of customer nodes that vehicle k is responsible for serving. The total safety loss of all vehicles during distribution can be expressed as

$F=d\Delta W^{2}\sum_{k=1}^{K} \sum_{i=1}^{n_{k}} \sum_{j=i}^{n_{k}} q_{j}[t_{_{(i-1)ik}}^{d}+\eta_{ik}+t_{ik}^{s}]$ (4)

## 2.4 Calculation of temperature control cost

Because different types of refrigerated food have different temperature control requirements, the appropriate temperature needs to be set based on the characteristics of the food to ensure that the quality of the food is maintained during transportation. According to the method of Ref. [20], the coefficient of performance (COP) is used to describe the conversion rate between energy and heat: $COP\text{=}\frac{\text{T}_{\text{L}}}{T_{H}-T_{L}}$, where $T_{H}$ is the ambient temperature outside, and $\text{T}_{\text{L}}$ is the target temperature. Both are thermodynamic temperatures, corresponding to absolute temperatures, denoted as T (K), and the unit is Kelvin (K). The relationship between the thermodynamic temperature T (K) and the Celsius temperature t is $T(K)=273+t(^{\circ}C)$. If the outside temperature $T_{H}$ is 30$^{\circ}C$(303 K) and the target controlled temperature is 10$^{\circ}C$(280 K), the COP values at different temperatures can be calculated. A refrigerated vehicle sets the proper refrigerated temperature based on the characteristics of milk. Table 1 lists the values of COP, $g_{w}$, and $\theta_{w}$ at different temperatures TL ($^{\circ}\text{C}$).

Table 1. The values of COP, $g_{w}$, and $\theta_{w}$ at different temperatures TL

| T_L_ | COP | $\theta_{w}$ | $Tf_{w}$ | $g_{w}$ | T_L_ | COP | $\theta_{w}$ | $Tf_{w}$ | $g_{w}$ |
| --- | --- | --- | --- | --- | --- | --- | --- | --- | --- |
| 10 | 14.15 | 1 | 4.84 | 30 | 4 | 10.65 | 1.33 | 6.44 | 39.9 |
| 9 | 13.43 | 1.05 | 5.08 | 31.5 | 3 | 10.22 | 1.38 | 6.68 | 41.4 |
| 8 | 12.77 | 1.11 | 5.37 | 33.3 | 2 | 9.82 | 1.44 | 6.97 | 43.2 |
| 7 | 12.17 | 1.16 | 5.61 | 34.8 | 1 | 9.45 | 1.5 | 7.26 | 45 |
| 6 | 11.63 | 1.22 | 5.90 | 36.6 | 0 | 9.10 | 1.55 | 7.50 | 46.5 |
| 5 | 11.12 | 1.27 | 6.15 | 38.1 |  |  |  |  |  |

Assuming that the outside temperature is 30$^{\circ}C$ and the cost of cooling to 10$^{\circ}C$ is one unit, i.e., $\theta_{w}$=1 (w=10$^{\circ}C$), then the cooling cost coefficient of 9$^{\circ}C$, $\theta_{w}$(w=9$^{\circ}C$), is 14.15/13.43=1.05. The cooling cost coefficients for other controlled temperatures can be calculated. The cost of temperature control per unit time at different temperatures can be obtained by solving the equation: $g_{w}=gf*Tf_{w}*\theta_{w}$, where $gf$ represents the fuel price, $Tf_{w}$ denotes the fuel consumed by a vehicle to achieve the controlled temperature of w per unit time during food distribution. Then, the total cost WC of temperature control for all vehicles during the distribution process is

$WC=\sum_{k=1}^{K} \sum_{i=1}^{n_{k}} \sum_{j=i}^{n_{k}} q_{j}[t_{_{(i-1)i}}^{d}+\eta_{ik}+t_{i}^{s}]g_{w}$ (5)

## 2.5 Vehicle fuel consumption and carbon emission calculation

The cost of carbon emissions during the cold chain distribution of food mainly includes the cost of fuel consumption for operating vehicles and controlling the temperature. In this model, the carbon emissions generated by fuel consumption during transportation are mainly calculated using the methodologies to estimate emissions from transport (MEET) model ^[23]^. The specific procedures are as follows. The carbon emission rate (g/km) of vehicle k on route section h of route (i, j) is

$\omega(v)_{ijkh}=\omega_{0}+\omega_{1}v+\omega_{2}v^{2}+\omega_{3}v^{3}+\omega_{4}/v+\omega_{5}/v^{2}+\omega_{6}/v^{3}$ (6)

where $\omega(v)_{ijkh}$ is the carbon emissions rate of an empty vehicle driving on a road without a slope at a speed of v ($v=v_{ijkh}$), and $\omega_{0},\omega_{1},\omega_{2},\omega_{3},\omega_{4},\omega_{5},\omega_{6}$ are correction factors, the values of which are dependent on the type of the loaded vehicle. The loading correction factors of the MEET model are

$LC_{ij}=\chi_{0}+\chi_{1}\gamma_{ij}+\chi_{2}\gamma_{^{ij}}^{2}+\chi_{3}\gamma_{^{ij}}^{3}+\chi_{4}v+\chi_{5}v^{2}+\chi_{6}v^{3}+\chi_{7}/v$ (7)

where $\gamma_{ij}$is the ratio between the real loading and the capacity on route (i, j), and $\chi_{0}\text{，}\chi_{1}\text{，}\chi_{2}\text{，}\chi_{3}\text{，}\chi_{4}\text{，}\chi_{5}\text{，}\chi_{6}\text{，}\chi_{7}$ are loading correction factors, the values of which depend on the type of the loaded vehicle. Thus, the carbon emission rate (kg/km) of vehicle k on route section h is $\text{cy}_{ijkh}=\omega(v)_{h}*LC_{ij}/1000$. According to ce=2.32 ^[17]^, the fuel consumption rate of vehicle k on route section h is $\text{fc}_{\text{ijkh}}=\text{cy}_{ijkh}\text{/ce}$; therefore, the carbon emissions cost of vehicle k on route (i, j) is

 (8)

The fuel consumption cost of vehicle k on route (i, j) is

 (9)

Meanwhile, the cost of carbon emissions due to temperature control during the distribution process is $\frac{WC}{gf}*ce*cf$.

## 2.6 Symbols and variables

$\text{N}\left\{\text{n}|\text{n}=0,1,2...\text{N}\right\}$: the set of all nodes in the cold chain logistics distribution network; 0 denotes the distribution center, $\text{N}`=\text{N}\backslash\left\{0\right\}$ denotes the set of customer nodes;

$\text{K}\left\{\text{k}|\text{k}=1,2...\text{K}\right\}$: the set of cold chain distribution vehicles;

Q: the maximum load capacity of vehicle (t);

$W\{w|w=1,2...W\}$: the set of controlled temperatures w;

c_1_: the fixed cost per vehicle;

c_2_: the labor and rent cost of a vehicle per unit time;

$\text{q}_{i}$: the demand of customer node i, (t), q_i_<Q;

$T_{ik}^{arrive}$: the time at which vehicle k arrives at node i, $T_{ik}^{leave}$the time at which the vehicle leaves node i;

$\eta_{jk}$: the waiting time if vehicle k arrives at node j in advance, $\eta_{jk}=ET_{j}-T_{jk}^{arrive}$ if $T_{jk}^{arrive}<ET_{j}$; otherwise,$\eta_{jk}=0$;

$t_{_{ijk}}^{\text{d}}$: the travel time of vehicle k from node i to node j;

$\text{t}_{_{i}}^{s}$: the unloading time needed when serving customer node i;

$[\text{ET}_{\text{i}},\text{LT}_{\text{i}}]$: the time window when customer i expects to be served;

$v_{ijkh}$: the velocity the distribution vehicle k on route h from node i to node j. Both $v_{c}$ and $v_{f}$ may be used;

$\text{p}$: the unit cost of transporting fresh food;

$\text{FC}_{ij}$: the fuel consumption cost of vehicle k on route (i, j);

$\text{CY}_{ij}$: the carbon emissions cost of vehicle k on route (i, j);

ce: the carbon emissions generated by 1 L of gas;

$cf$: the unit cost of carbon emissions;

x_kw_: a dummy variable (0 and 1): x_kw_ =1 if vehicle k is used at a controlled temperature of w; otherwise, x_kw_ =0;

y_ikw_: a dummy variable (0 and 1): y_ikw_ =1 if vehicle k with a controlled temperature of w serves customer node i; otherwise, y_ikw_ =0;

z_ijkw_: a dummy variable (0 and 1): z_ijkw_ =1 if vehicle k with a controlled temperature of w travels on route (i, j); otherwise, z_ijkw_ =0.

## 2.7 Mathematical model

The optimization goals of this paper are the minimum economic cost of logistics distribution for distribution enterprises and the minimum food safety loss cost. The time-dependent vehicle routing problem with time windows (TDVRPTW) optimization model constructed for cold chain food distribution is as follows:

$\begin{matrix} \min\sum_{k\in\text{K}} x_{kw}\text{c}_{1}+c_{2}(\sum_{k\in K} \sum_{i\in N} \sum_{j\in N} z_{ijkw}t_{_{ijk}}^{d}+\text{ }\sum_{i\in N`} t_{i}^{s}+\sum_{k\in K} \sum_{j\in N`} y_{jkw}\eta_{ik})+ \\ \sum_{k\in K} \sum_{i\in N} \sum_{j\in N} \sum_{h\in H} z_{ijkw}(CY_{ij}+FC_{ij})+WC+\frac{WC}{gf}*ce\text{*cf}+\text{p}*F \end{matrix}$ (10)

s.t.$\text{ }\sum_{k\in K} y_{jkw}=1,\forall j\in N'$ (11)

$\sum_{i\in N} z_{ijkw}=\sum_{j\in N} z_{ijkw},\forall k\in K$ (12)

$\text{t}_{_{ijk}}^{d}=\sum_{h=1}^{H} t_{_{ijkh}}^{d},\forall i\in N,j\in N,k\in K$ (13)

$\sum_{k\in K} n_{k}=n$ (14)

$\sum_{j=1}^{n_{k}} q_{j}\leq Q$，$\forall k\in K$ (15)

$\sum_{j\in N`} z_{0jkw}\leq1,\forall k\in K$ (16)

$ET_{i}\leq T_{ik}^{arrive}+\eta_{ik}\leq LT_{i},\forall i\in N,k\in K$ (17)

$T_{ik}^{arrive}+\eta_{ik}+t_{i}^{s}=T_{ik}^{leave}$，$\forall i\in N,k\in K$ (18)

$T_{jk}^{arrive}=T_{ik}^{leave}+t_{ijk}^{d}$，$\forall i,j\in N,k\in K$ (19)

$\text{x}_{kw}\in\{0,1\},y_{ikw}\in\{0,1\},z_{ijkw}\in\{0,1\}\text{ }$ (20)

Eqn. (10) is the objective function that minimizes the economic cost of logistics distribution and the cost of food safety losses. The economic cost of distribution includes the fixed cost of the vehicles, labor costs, and the fuel consumption and carbon emissions generated by driving and controlling the temperature during transportation. The labor cost incorporates the cost of labor during the vehicle driving time, the waiting time, and the service time. Eqn. (11) requires that each customer must be served exactly once. Eqn. (12) indicates that the vehicle arrives at and leaves from the same node. Eqn. (13) shows the relationship between the travel time on the entire route and the travel times on sections of the route. Eqn. (14) requires that all customer nodes are served with no omissions. Eqn. (15) gives the vehicle capacity constraints. Eqn. (16) requires that each vehicle is only used once. Eqn. (17) gives the time window constraints. Eqns. (18-19) express the relationships between the arrival time, waiting time, service time, and leaving time at each customer node. Eqn. (20) gives the variable value constraints.

**3. The design of the improved ant colony algorithm**

The fresh food distribution routing optimization problem with time windows is an extension of the VRP with time windows problem. The problem becomes more complex when considering traffic congestion; it is an NP-hard problem. The ACA exhibits good characteristics, such as a self-organizing capability, a positive feedback mechanism, and a parallel search ability, which enable it to more effectively solve complex combinatorial optimization problems and to be widely used in many fields. Based on the features of the model in this paper, an IACA is designed to solve the model. The specific procedures are as follows.

Procedure 1: Initialize the relevant variables. Set the number of iterations as NC=1 and the maximum number of iterations as NCmax. Place M ants at the distribution center. Let the initial pheromone matrix $\tau_{ij}(NC) =c$, where c is a constant. Compute the distance matrix between nodes. Set the global optimality to bestcost and the current ant as m=1.

Procedure 2: Construct feasible solutions.

Step 1. With respect to ant m, place all unvisited customer nodes into the set tovisit_m_. Set the vehicle number factor vn=1.

Step 2. Calculate the transition probability of each node that can be chosen by the ant.

(21)

where $\tau_{ij}$ is the pheromone concentration; $\eta_{ij}$ denotes the visibility $\eta_{ij}=1/d_{ij}$; the saving matrix $U_{ij}$ is used as the prior information to guide the ant’s searching, where $U_{ij}=d_{i1}+d_{j1}-d_{ij}$; to address the priority when serving customer nodes, waiting time $\eta_{jk}$ is introduced; to avoid a zero probability, which disables the ant from choosing the next node, appropriate measures have been taken; and represent the relative importance of the pheromone concentration, visibility, saving matrix factor, and waiting time factor, respectively.

Step 3. Find node j. To prevent the algorithm from falling into local optimization and premature stagnation, the strategy of state transition is improved by the roulette wheel method according to Ref. [24]. The specific operation is as follows:

$\text{j=}\left\{\begin{matrix} \mathop{\mathrm{argmax}}\limits_{j\in tovisit} \{P_{ij}^{m}\},\text{ if }R_{1}\leq R_{0} \\ \text{Randomly selected point j (}j\in tovisit\text{)based on roulette wheel method},if\text{ }R_{1}>R_{0} \end{matrix}\right.$ (22)

where argmax is the maximum operation; R_1_ is a random number in [0,1]; and R_0_ is a constant in [0,1]. Find the corresponding node j. Ant m travels to customer node j if Q and the time window constraints are satisfied; otherwise, the ant returns to the distribution center, sends out a new vehicle, vn=vn+1, and repeats Step 1 until all nodes have been visited.

Step 4. Compute the departure time of each vehicle as ant m from the distribution center.

Step 5. m=m+1. Repeat Step 1 if m<M.

Procedure 3: Compute the objective function values of the routes. Calculate the objective function value O(R_m_)_NC_ of the searching route R_m_ for each ant based on Eqn. (11). If $\text{O}\left(\text{R}_{\text{m}}\right)_{\text{NC}}\leq best\cos t$, $best\cos t=\text{O}\left(\text{R}_{\text{m}}\right)_{\text{NC}}$. Then, NC=NC+1.

Procedure 4: Update the global pheromones. Upgrade the pheromone values of the routes traversed by the optimal ant.

$\tau_{ij}(NC+1)=\rho\tau_{ij}(NC)+\Delta\tau_{_{ij}}^{m}(NC)$ (23)

$\Delta\tau_{ij}^{m}=\left\{\begin{matrix} Q/O(R_{m}),\text{ Ant m travels on the road(i,j),} \\ 0,otherwise \end{matrix}\right.$ (24)

where $\rho$ is the pheromone volatility, $\rho\in[0,1]$; $\Delta\tau_{_{ij}}^{m}$ is the pheromone increment; Q is a constant; and $O(R_{m})$ is the total distribution cost generated by this ant route search. To ensure that the pheromones accumulated on each road are not too excessive or sparse, the pheromone concentration $\tau_{ij}$ on all routes is set within $[\tau_{\min},\tau_{\max}]$.

Procedure 5: Determine if the algorithm terminates. Check if NC reaches NCmax. Repeat Procedure 2 if it has not; otherwise, the algorithm terminates.

**4. Example simulation**

## 4.1 Experimental setup

The test example data were from the Solomon’s international standard VRPTW databases ^[25]^. Considering various factors of fresh food distribution, we used the data of the type C (concentrated distribution), type R (random distribution), and type RC (mixed distribution) examples of the international standard VRPTW database. In each example, there are 100 customer points and one distribution center. To be more realistic, the above cases were modified as follows. A fresh milk production enterprise distribution center needed to distribute milk to 100 customer points within the city. The initial working time was set as 6:00 am, with a corresponding time point of 0. According to urban traffic patterns, the time periods of 7:00 am to 9:00 am and 17:00 pm to 19:00 pm were set as traffic congestion hours, and the remaining time periods were assumed to be normal traffic hours. The driving speed during normal traffic hours (v_f_) was 60 km/h, and that during traffic congestion hours (v_c_) was 30 km/h.

The algorithm was programmed with MATLAB R2016a and executed on a computer with 8 GB of RAM and a 1.90-GHz CPU. According to the methods in Refs. [11, 17, 20], the variable parameters were set as follows. For fresh milk, d=4*10-5, and p=1000 yuan/t. The basic parameters of the vehicle are Q=1000 units, one unit load is 2 kg, the vehicle capacity is 2 tons, c1=150 yuan/vehicle, and c2=0.5 yuan/min. Let $\text{w}$=10$^{\circ}C$, $\text{W}_{\min}=0^{\circ}C$, and the outside temperature be 30$^{\circ}C$. The corresponding carbon emission rate coefficients are $\omega_{0}=110,\omega_{1}=0,\omega_{2}=0,\omega_{3}=3.75*10^{-4},$ $\omega_{4}=8702,\omega_{5}=0,\omega_{6}=0;$ and the corresponding loading correction factor coefficients are $\chi_{0}=1.27,\chi_{1}=0.0614,\chi_{2}=0,\chi_{3}=-0.0011\text{，}\chi_{4}=-0.00235,$ $\chi_{5}=0,\chi_{6}=0,\chi_{7}=-1.33$, $gf=6.2yuan/L$, and $cf=0.0\text{528yuan}/\text{kg}$. The values of the parameters in the algorithm were taken from Ref. [26], as shown in Table 2.

Table 2. Ant colony algorithm parameters

| **Parameter** | M | NCmax | $\alpha$ | $\beta$ | $\gamma$ |  | Q |
| --- | --- | --- | --- | --- | --- | --- | --- |
| **Value** | 40 | 400 | 1 | 3 | 3 | 2 | 100 |

## 4.2 Experimental results

### 4.2.1 Large-sized data example

The example was calculated with the dataset RC208. The running time was 310.3 seconds, and the overall distribution cost was 6464.5 yuan. A total of six vehicles were dispatched for delivery. The cost of the labor and vehicles was 2610.2 yuan. The cost of food safety losses was 660.3 yuan. The fuel consumption costs generated by temperature control and transportation were 1041.5 and 2090.7 yuan, respectively. The carbon emissions cost was 61.8 yuan. The specific routing optimization plan is presented in Table 3. In Table 3, SN denotes the number of the vehicle, VR represents the driving route of the vehicle (0 denotes the distribution center, and 0-100 denote customer nodes), and VAT represents the time at which the vehicle arrives at each customer node.

Table 3. Vehicle route optimization results of the example with the RC208 dataset

| SN | VR | VAT |
| --- | --- | --- |
| 1 | 0-1-2-3-4-5-45-6-7-8-46-78-9-13-10-14-17-0 | 349.92-388-408.44-428.44-443.82-458.82-471.82-489.10-502.10  -517.10-530.10-566.10-597.20-612.20-629.27-646.34-661.34-741.96 |
| 2 | 0-11-12-15-16-18-19-21-23-22-57-52-86-20-49-24-48-25- 77-58-59-87-74-84-26-32-0 | 0-33.52-48.52-66.33-94-218-233.38-248.77-260.77-280.96-305.11-322.18-339.39-391-404-419-439.77-456.77-478.93-499.56-520.74-535.21-554.16-602.24-650.25-668.25-770.41 |
| 3 | 0-27-29-31-28-30-33-63-85-51-34-50-35-36-37-38-39- 41-40-43-42-54-96-0 | 0-57.01-74.01-88.01-110.81-128.81-150.47-196.09-209.69-228.24-342-365-425.24-437.25-450.25-463.85-475.85-491.24-511.43-526.43-541.44-571.44-587.44-602.96 |
| 4 | 0-44-72-71-67-62-95-47-73-79-53-88-55-70-56-91-60- 100-68-0 | 0-49-85.57-115.97-152.05-176.19-197.41-262.00-287.00-305.94-338.75-357.69-420-440.29-487.45-504.53-553 -580.46-601.86-611.86 |
| 5 | 0-61-64-83-65-82-98-69-66-75-76-89-80-0 | 78.38-110-190-208.06-227.28-245.82-264.88-279.88-404-462.16-527.61-547.51-604.03-611.65 |
| 6 | 0-81-90-99-92-94-93-97-0 | 236.96-249-275.28-301.56-339.72-356.12-373-449.37-497.53 |

The following observations can be made from the calculations. (1) The algorithm in this paper can yield optimal route planning in a relatively short time. (2) By combining the VR and VAT, it can be seen that due to the constraints of city traffic congestion, customer service time windows, and vehicle capacity, obvious differences exist between the cold chain logistics vehicle distribution routes. Vehicle 2 served the most customer nodes, at 25; Vehicle 6 served the least number of customer nodes, at only 7. The reason is that the time window of each node is different. Vehicle route planning must ensure a minimal cost while satisfying the time windows of the customer nodes. (3) From the VAT, it can be seen that Vehicles 2, 3, and 4 depart at time 0 from the distribution center to deliver, and the other vehicles depart at different times. If departing at time 0, a vehicle will incur a relatively large cost of waiting due to early arrival, suggesting that it is necessary for logistics enterprises to consider time dependence when planning routes. They should scientifically plan the routes based on real situations, such as the road network conditions and the customer node time windows, and comprehensively consider the costs, such as the temperature control and fuel consumption costs during transportation. (4) According to the VAT, Vehicle 6 completely avoided the congestion periods. Vehicles 3, 4, and 5 only entered the congestion period of the morning rush hour but did not enter the evening rush hour. Vehicle 1 experienced the evening rush hour congestion but not the morning congestion. This result indicates that the method proposed in this paper can help to appropriately avoid the traffic congestion periods and increase the distribution efficiency of vehicles.

### 4.2.2 Example simulation at different controlled temperatures

In this example, the temperature in the refrigerated vehicles is assumed to vary within a certain range, and other characteristics, such as the time windows and geographical factors, remain constant. The temperature control cost and food safety losses change during transportation, affecting the logistics distribution costs and lowering the safety and value of the food. The temperature sensitivity analysis used the dataset RC208, with milk distribution as an example. The temperature in the fresh food distribution vehicles was controlled within the range from 0$^{\circ}C$ to 10$^{\circ}C$. The sensitivity relationships between the refrigerated temperature, temperature control cost, safety loss cost, and logistics cost are illustrated in Figures 3 and 4.

Fig. 2. The relationship between the safety loss and temperature control costs

Fig. 3. Sensitivity analysis of the distribution cost and refrigerating temperature

As seen from Figure 2, as the refrigerated temperature gradually decreased from 10$^{\circ}C$, the safety loss cost gradually declined, but the temperature control cost increased accordingly. At a refrigerated temperature of 10$^{\circ}C$, the temperature control cost during milk distribution was a minimum, but the food safety loss was a maximum; on the contrary, when the refrigerated temperature was 0$^{\circ}C$, the safety loss of milk was zero because no microorganisms grew, but the temperature control cost was the highest. Thus, the safety loss had a paradoxical relationship with the temperature control cost. According to Figure 3, as the refrigerated temperature decreased, the total logistics distribution cost decreased. At a refrigerated temperature of 5$^{\circ}C$, the logistics cost was the lowest. Then, as the refrigerated temperature decreased, the logistics cost increased, suggesting that 5$^{\circ}C$ is preferable to other refrigerated temperatures; thus, 5$^{\circ}C$ is the optimal refrigerated temperature for transporting milk. Neither increasing nor decreasing the temperature can optimize the total logistics distribution cost.

### 4.2.3 Optimization simulation on examples with different customer distributions

Different types of examples were used to solve the milk cold chain transportation problem. The controlled refrigerated temperature was 10$^{\circ}C$. Each example was solved 10 times, and the average value was taken as the final result. Table 4 shows the experimental results, where EX represents the type of example, TC denotes the total distribution cost, TMC denotes the fixed costs of the vehicles and labor, WC is the fuel consumption cost generated by temperature control, FC is the fuel consumption cost during driving, CF is the carbon emission cost generated by driving and temperature control, SC is the safety loss cost, and VN is the number of vehicles.

Table 4. Simulation results of examples with different customer distributions

| **EX** | **TC** | **TMC** | **TMC/TC** | **WC** | **FC** | **(WC+FC)**  **/TC** | **CF** | **CF/TC** | **SC** | **VN** |
| --- | --- | --- | --- | --- | --- | --- | --- | --- | --- | --- |
| C103 | 16812.4 | 9514.3 | 56.59% | 2812.9 | 3385.2 | 36.87% | 122.46 | 0.73% | 977.6 | 24 |
| C104 | 16075.5 | 9190.1 | 57.17% | 2479.4 | 3308.3 | 36.00% | 114.35 | 0.71% | 983.4 | 21 |
| R203 | 10261.7 | 4260.9 | 41.52% | 1255.5 | 3967.7 | 50.90% | 103.20 | 1.01% | 674.4 | 11 |
| R204 | 8677.8 | 3179.3 | 36.64% | 1128.7 | 3591.6 | 54.40% | 91.28 | 1.05% | 686.9 | 8 |
| RC207 | 8134.2 | 3210.3 | 39.47% | 1183.2 | 2958.6 | 50.92% | 81.83 | 1.01% | 700.3 | 8 |
| RC208 | 6464.5 | 2610.2 | 40.93% | 1041.5 | 2090.7 | 48.96% | 61.8 | 1.09% | 660.3 | 6 |

A. Vehicle route planning and optimal value iteration of example C103

B. Vehicle route planning and optimal value iteration of example R204

C. Vehicle route planning and optimal value iteration of example RC207

(x: abscissa km; y: ordinate km; NC: the number of iterations; TC: the total cost)

Fig. 5. Vehicle route planning for examples with different distribution types

The following can be seen from Figure 5 and Table 4. (1) According to Figure 5, the algorithm reached the optimal solution and stability within 400 iterations when solving different types of examples, indicating that the algorithm can effectively solve the model in this paper and has a good convergence property. (2) The type C example had the highest total logistics distribution cost, vehicle use and labor cost, safety loss cost, temperature control cost, and number of vehicles used among all types, but its fuel consumption cost was lower than that of the other types. This difference is mainly because the customer nodes of the type C distribution were mainly concentrated at several regions, and the driving distances between the customer nodes were relatively short, which lowered the fuel consumption cost. Nevertheless, the time windows of the customers were relatively narrow. In the Solomon dataset, the service time at a customer node of the type C model was 90 minutes, as opposed to the 10-minute service time at the R and RC type customer nodes. Therefore, a higher requirement was placed on vehicles to arrive at customer nodes on time and satisfy the time windows. As a result, the vehicles could only serve fewer customers and required a longer total driving time, leading to a higher overall distribution cost. (3) Compared to type C, types R and RC had a lower total distribution cost, vehicle use and labor cost, temperature control cost, safety loss cost, and number of vehicles. These differences can mainly be attributed to the 10-minute service time at the customer nodes of these two types. The time window requirements of the customers were relatively loose, so the vehicles could visit many customers, and the total driving times were relatively short. (4) For all types of examples, the TMC made up a relatively high proportion in the total distribution cost, accounting for approximately 40% of the total. For type C, the proportion was greater than 50%, indicating that the cost of vehicle usage is one of the major sources of the logistics distribution costs. Because driving time is the main factor, to lower the logistics costs, the primary task should be reducing the total transportation time. (5) The fuel consumption costs generated by temperature control and driving also accounted for a relatively high proportion of the logistics costs, accounting for 36% of the total logistics costs for type C and approximately 50% for types R and RC. This result suggests that the fuel consumption due to temperature control and driving is also a major source of the logistics costs. Because the main factors are the driving time and the controlled refrigerated temperature, to reduce the logistics costs, the main task is to control the transportation time and properly adjust the refrigerated temperature. (6) The proportion of carbon emissions was very low, accounting for only 0.7-1.1% of the total costs, implying that from an economic perspective, the carbon emissions cost caused by the current carbon tax can hardly effectively promote energy savings and emissions reductions among logistics enterprises.

### 4.2.4 Simulation under different congestion levels

On the basis of Section 4.2.1, with the other parameters in the algorithm remaining constant and $v_{f}$ set to 60 km/h, the dataset RC208 was used with different congestion driving speeds (40 km/h, 30 km/h, 20 km/h). For each condition, 10 calculations were carried out, and the average value was taken as the final result. The optimization results obtained are shown in Table 5.

Table 5. Simulation results of different congestion degrees

| **V_c_** | TC | TMC | **WC** | **FC** | **CF** | SC | **VN** |
| --- | --- | --- | --- | --- | --- | --- | --- |
| 40 | 6686.4 | 2599.7 | 1029.7 | 2006.9 | 59.9 | 647.8 | 6 |
| 30 | 6464.5 | 2610.2 | 1041.1 | 2090.7 | 61.8 | 660.3 | 6 |
| 20 | 6686.4 | 2776.8 | 1102.3 | 2103.1 | 63.3 | 640.9 | 7 |

The following observations can be obtained from Table 5. (1) As the congestion level increased, the driving speed of the vehicles decreased, while the total distribution cost, the vehicle use and labor cost, the fresh food safety loss cost, and the fuel consumption cost due to temperature control and driving all increased to different extents, indicating that these costs are positively correlated with the congestion condition. (2) Increasing the congestion coefficient led to increases in the fuel consumption and carbon emissions, suggesting that the congestion condition can influence the “green” level of the cold chain logistics distribution route. The food safety loss cost also increased, mainly because the driving time was affected, indicating that traffic congestion can influence the vehicle speed and thus affect the vehicle route planning.

### 4.2.5 Comparative analysis of different algorithms

The fresh food cold distribution optimization problem is solved by the IACA, conventional ACA ^[27]^, and tabu search algorithm (TS) ^[28]^. The starting time of vehicles in the ACA and TS is 0. There is no waiting factor or saving matrix in the ACA, unlike the IACA. We used the data of C104, R204, and RC204 for comparison. Note that all of the algorithms are well tuned to achieve the best performance. The results obtained are shown in Table 6, where CPUT is the computation time.

Table 6. Calculation results of the three algorithms

| EX | IACA | | ACA | | TS | |
| --- | --- | --- | --- | --- | --- | --- |
|  | TC | CPUT | TC | CPUT | TC | CPUT |
| C104 | 16075.5 | 379.7 | 16581.6 | 361.3 | 16818.7 | 351.3 |
| R204 | 8677.8 | 371.3 | 8901.5 | 359.4 | 9216.3 | 352.1 |
| RC208 | 6464.5 | 369.9 | 6701.3 | 355.7 | 6948.2 | 347.9 |

The following observations can be obtained from Table 6. (1) The distribution cost of the IACA is lower than that of the two conventional algorithms. (2) In terms of computing time, compared with the two conventional algorithms, the IACA in this paper mainly considers the starting time calculation and the improvement of the state transition probability, but the time consumed by the three algorithms is similar, and all of them can obtain the optimal results in relatively less time. (3) Although the calculation time is relatively fast in the TS, it is highly dependent on the initial value, and the algorithm suffers from convergence problems.

**5. Conclusions**

Traffic congestion has become a common phenomenon in cities, which prolongs the driving time during cold chain logistics transportation and significantly increases both temperature control costs and food safety losses. Meanwhile, the generated carbon emissions pollute the atmosphere of cities. With respect to the fresh food cold chain distribution optimization problem under time-varying road network conditions, this paper constructs a TDVRPTW model considering temperature control and food safety and designs a corresponding ACA to solve the model. The experimental results suggest the following. (1) Logistics enterprises should appropriately plan distribution routes based on real situations, such as the city road network, customer distribution, time windows, properties of fresh food, and outside environment, to effectively avoid traffic congestion periods and reduce the total logistics costs. (2) The temperature control cost has a paradoxical relationship with the food safety loss cost. One should jointly consider the food characteristics and the outside environment to appropriately control the refrigerated temperature, thus reducing the total distribution cost. (3) The designed ACA can effectively solve the problem in this paper, with a good convergence. (4) Currently, the carbon tax in China is relatively low, so the present carbon trading price does not notably influence the vehicle routing scheduling of logistics enterprises. Therefore, the government should properly increase the carbon tax to effectively incentivize the transportation industry to save energy and reduce emissions.

Limited by our capabilities, we only study the cold chain transportation of a single type of product with a single type of vehicle, and we only consider recurring traffic congestion rather than deeply analyzing occasional traffic conditions. The next step of research should focus on the cold chain logistics transportation VRP with respect to multiple types of vehicles and products.

**Acknowledgments:** The authors would like to thank the anonymous reviewers for their remarkable comments and the great support of the National Natural Science Foundation of China (No. 71972069).

**Author Contributions:** Zhao Zhixue contributed to data analysis and writing the manuscript. Li Xiamiao contributed equally to this work and designed the empirical analysis. Zhou Xiancheng organized the manuscript and revised the analysis.

**Conflicts of Interest:** The authors declare no conflict of interest.

References

[1] Osvald A, Stirn L Z. A vehicle routing algorithm for the distribution of fresh vegetables and similar perishable Food. Journal of Food Engineering，2008,85(2):285-295．

[2] Chen H K，Hsueh C F，Chang M S． Production Scheduling and Vehicle Routing with Time Windows for Perishable food products．Computers ＆ operations research，2009，36(7) : 2311-2319

[3] Tiwari A, Chang Peichann. A block recombination approach to solve green vehicle routing problem. International Journal of Production Economics, 2015, 164:379-387.

[4] C.D.Tarantilis, C.T.Kiranoudis. A meta-heuristic algorithm for the efficient distribution of Perishable foods. Joumal of Food Engineering，2001,50:1-9.

[5] P.Amorim et al. A rich vehicle routing Problem dealing with Perishable food:a case study. Springer, 2014, 22:489-508.

[6] Xiangguo, M.; Tongjuan, L.; Pingzhe, Y.; Rongfen, J. Vehicle Routing Optimization Model of Cold Chain Logistics Based on Stochastic Demand. J. Syst. Simul. 2016, 8:1824-1832.

[7] Lan H, HE Q F, Bian Z, Jin Z H. Distribution routing optimization of cold chain logistics with consideration of road traffic conditions. Journal of Dalian Maritime University. 2015,11(41):67-74.

[8] Li N, Wang S B. Production and distribution optimization model for perishable products in uncertain demand. Application Research of Computer. 2015,11(41):67-74.

[9] Ma X G, Liu T J, Yang P Z. Vehicle Routing Optimization Model of Cold Chain Logistics Based on Stochastic Demand. Journal of System Simulation. 2016, 8:1824-1832.

[10] Shao J P, Cao Q, Shen M Y, et al. Research on multi-objective optimization for fresh agricultural products VRP problem. Industrial Engineering and Management, 2015, 20(1): 122- 127.

[11] KANG K, HAN J, PU W, et al. Optimization research on cold chain distribution routes considering carbon emissions for fresh agricultural products. Computer Engineering and Applications, 2019, 55（2）：259-265.

[12] Zhang Y M, Li Y M, Liu H O. Research on VRP Optimization of Multi-type Vehicle Cold-chain Logistics With Satisfaction Constraint. [Statistics and Decision](http://www.baidu.com/link?url=L7I_yqfA_1X0QFrPVuEdTo4oZkPFSfSj9MvhBhW53KwrL5n5cgWP2k3yEo8ySB_9l86LtD0dEulwZxiOF8V979m0FBRmAFPWZ-lowl5wm3-rw5aOpLJw1e3r07pgRdm9), 2019,4:176-181.

[13] Ma X L,Wang S Y, Liu X B, et al. Integrated optimization of production scheduling and distribution for perishable food products in two-echelon supply chain. Industrial Engineering and Management, 2017, 22(2): 46-52.

[14] Devapriya P, Ferrell W, Geismar N. Integrated production and distribution scheduling with a perishable product. European Journal of Operational Research, 2016, 259(3):906-916.

[15] Wu Y, Ma Z J. Time-dependent production delivery problem with time windows for perishable food. Systems Engineering-Theory & Practice, 2017,37(01): 172-181.

[16] Wang Y, Zhang J. Optimization Method Study of Fresh Good Logistics Distribution Based on Time Window and Temperature Control . Control and Decision [Online]. Available: DOI: 10.13195/j.kzyjc.2018.1662

[17] Liu C S,Zhou X C, Sheng H Y, Luo L. Research on TDVRPTW of Fresh E-commerce Distribution: Considering both Economic Cost and Environmental Cost. Control and Decision [Online]. Available: <https://doi.org/10.13195/j.kzyjc.2018.1582>.

[18] Wang S Y, Tao F M, Shi Y H, Wen H L. Optimization of Vehicle Routing Problem with Time Windows for Cold Chain Logistics Based on Carbon Tax. Sustainability ,2017, 9, 694-717

[19] ZWIETERING M H，DE WIT J C, Notermans S. Application of predictive microbiology to estimate the number of Bacillus cereus in pasteurized milk at the point of consumption. International Journal of Food Microbiology,1996, 30(1):55-70.

[20] Xie R H, Huang X R. Emergency Mechanism of Food Logistics for Mega Emergency Events. Bei Jing, CHN, Social Sciences Academic Press, 2013, pp. 67-105.

[21] Xiao Y, Konak A. The heterogeneous green vehicle routing and scheduling problem with time-varying traffic congestion. Transportation Research Part E. 2016, 88:146-166.

[22] Soysal M, Bloemhof-Ruwaard J M, Bektas T. The time-dependent two-echelon capacitated vehicle routing problem with environmental considerations. International Journal of Production Economics,2015,164:366-378.

[23] Hickman AJ. Methodology for calculating transport emissions and energy consumption. Cold Starts, 1999.

[24] Fang W T, Ai S Z. Wang Q. Research on Cold Chain Logistics Distribution Path Optimization Based on Hybrid Ant Colony Algorithm. Chinese Journal of Management Science, 2019, 27(11):108-115.

[25] http://w.cba.neu.edu/~msolomon/problems.htm

[26] Li L, Liu S X, Tang J F. Improved ant colony algorithm for solving vehicle routing problem with time windows. Control and Decision, 2010, 25(9): 1379-1383.

[27] Cordeau J F, Gendreau M, Laporte G, et al. A guide to vehicle routing heuristics. Journal of the Operational Research Society, 2002,53(5):512- 522.

[28] P. Devapriya, W. Ferrell, N. Geismar. Integrated production and distribution scheduling with a perishable product. European Journal of Operational Research, 2016,259 (3):906-916.

# Data Availability statement: All data supporting the findings of this study are from previously reported studies and datasets, which have been cited. The processed data are available in the supplementary files or from the corresponding author upon request.
