# Supplementary material for: Optimization of transportation routing problem for fresh food in time-varying road network: Considering both food safety reliability and temperature control
Source: PLoS One. 2020 Jul 27;15(7):e0235950. doi: 10.1371/journal.pone.0235950 (PMC7384659; doi:10.1371/journal.pone.0235950)
Supplement: Attachment — Submitted filename: review comments-nie.docx [file pone.0235950.s002.docx]

This paper proposed a time-dependent vehicle routing problem with time windows (TDVRPTW) optimization model for cold chain food distribution. In the proposed model, the authors consider three new factors (e.g., food safety, energy consumption and carbon emission) for solving the vehicles routing problem. This paper obtains some meaningful conclusions by the case studies.

The reviewer feels that the topic has certain theoretical significance, and the paper can clearly state its contributions. However, there are some problems in this paper which make the proposed model is not right or clear. The description of parameters is confusion and inconsistent. Therefore, the reviewer suggests that this paper should be revised carefully. The details of comments are below.

1. In the section 2.2, the paper explains the relationship between the driving speed and the departure time. The travel time function is formulated. However, in the following section, the travel time function is useless because the paper partition the route into a sufficiently short section and the vehicle speed on this short section is set to be constant.

2. In the section 2.3, $W_{min}$ is the temperature at which no microorganisms grow (Celsius) but it is not the minimum value of controlled temperature $w$. if the condition $w<W_{min}$ occurs, the formula (3) will get the wrong result that the food safety loss increases with the decrease of $w$.

3. In the formula (4), $t_{\left( i-1 \right)ik}^{d}$ is not right that leads to the wrong formula (4). The formula (5) also has the same mistake.

4. In the section 2.4, the units of $T_{H}$ and $T_{L}$ should be clear, Celsius or Kelvin (K).

5. In table 1, the value of COP is not right if $T_{H}=30℃$.

6. Why assume $\theta_{w}=1$ if $T_{H}=30℃$ and $T_{L}=10℃$?

7. In formula (5), why is the linear relationship between the customer demand and temperature control cost? As we know, the customer demand actually affects the temperature control cost. But the relationship is absolutely not linear. For example, the temperature control cost for transporting one hundred commodities is absolutely not 100 times that for transporting one commodity.

8. In formula (8) and (9), the product of $v_{ijkh}t_{ijkh}^{d}$ should be equal to $\vartheta$. Why not use $\vartheta$ instead of $v_{ijkh}t_{ijkh}^{d}$ to simplify the formula (8) and (9).

9. In the section 2.7, what is the meaning of $p*F$ in formula (10)?

10. Formula (12) is wrong. It cannot ensure that the vehicle arrives at and leaves from the same node.

11. Formula (18) and (19) cannot reflect the time window constraints properly. For example, if $T_{ik}^{arrive}>ET_{i}$, the waiting time $\eta_{ik}$ still exists that means the vehicle has to wait for a while even it arrives within the time window.

12. In the proposed model, it lacks the constraints for $y_{jkw}$ and $z_{ijkw}$. It means that if $z_{ijkw}=1$, $y_{jkw}$ must be 1. Whereas, if $y_{jkw}=1$, $z_{ijkw}$ can be 1 or 0.

13. In the proposed model, constraints that vehicles must return the distribution center are omitted.

14. In section 4.2.2, there is no figure 4.

15. The discussion for figure 2 is not appropriate because there is no temperature coordinate in figure 2.

16. Figure 5 should be Figure 4.

17. $5℃$ is the optimal refrigerated temperature observed in figure 3. Why not set the controlled refrigerated temperature to $5℃$ in the section 4.2.3.

18. In this paper, several variables are not defined before they are used (e.g., $d$, $a$, $v_{c}$, $v_{f}$, $ce$ and etc.) and some variable definitions are duplicated (e.g., $\vartheta$ and $h$, $k$ and $t$). A lot of variables are higher than the text and not italic. The subscripts of some variable are wrong (e.g. $TL$, $t_{\left( i-1 \right)i}^{a}$ ). The authors should be check all paper carefully and correct all mistakes.
