# Supplementary material for: Optimization of transportation routing problem for fresh food in time-varying road network: Considering both food safety reliability and temperature control
Source: PLoS One. 2020 Jul 27;15(7):e0235950. doi: 10.1371/journal.pone.0235950 (PMC7384659; doi:10.1371/journal.pone.0235950)
Supplement: Attachment — Submitted filename: PLOS ONE-Response-to-Reviewers.doc [file pone.0235950.s003.doc]

**Original Manuscript ID:** PONE-D-20-08429

Original Article Title: **“**Optimization of transportation routing problem for fresh food in time-varying road network: considering both food safety reliability and temperature control**”**

**To:** PLOS ONE Editor

**Re:** Response to reviewers

Dear Editor,

Thank you for allowing a resubmission of our manuscript, with an opportunity to address the reviewers’ comments.

We are uploading (a) our point-by-point response to the comments (below) (response to reviewers), (b) an updated manuscript with yellow highlighting indicating changes, and (c) a clean updated manuscript without highlights (PDF main document).

Best regards,

**Zhao Zhixue**

**Reviewer#1, Concern # 1:** Some statements/symbols seem unprofessional, and nomenclature is confusing., the N={0,1,2...N} should be corrected as N={0,1,2...n} ; and the difference between a set and a function, etc.

**Author response:** Thank you for your constructive and helpful suggestion. Some statements/symbols have been revised. All variables and sets names are standardized in section 2.2. For example, : Set of all nodes in the cold chain logistics distribution network; 0 denotes the distribution center, denotes the set of customers nodes.: Set of cold chain distribution vehicles.: Set of controlled temperatures *w*. $\mathbf{T}=\{T_{1},T_{2}\text{，}...\text{，}T_{m}\}$: Set of the time period throughout the day, *m* is the total number of time periods.

In section 2 and 3, we have updated normalized statements, such as *COP,$W_{min}$,$W_{O},W_{L}$.*

**Reviewer#1, Concern # 2:** Some figure format and quality are poorly provided. For example, the Fig. 5 A.

**Author response:** Thank you for your constructive and helpful suggestion. All the figures have been replaced as you required. We have uploaded the original picture to the attachment for the editorial board's review。

**Reviewer#1, Concern # 3:** The author only considers that the vehicle arrives early at customer node. However, In reality, the vehicle may be late. That is to say, the arrive time of vehicle exceeds the customer time window upper limit.

**Author response:** Thank you for your constructive and helpful suggestion. I updated the method to deal with the customer point window const raint in section 2.1: There is also a service time-window requirement. A vehicle can reach a client point to provide service at an earlier or later time but then will pay a certain penalty.

I have solved the customer point time window constraint by increasing the penalty cost in section 2.7:

**2.7 Vehicle penalty cost**

This penalty cost is incurred by the vehicle as a result of violating the client’s time window during the distribution process. Client *i* accepts service outside the time window, but there is a penalty cost for failing to provide service within the appointed time, and the total penalty cost *PC* equals to

(13)

: Time window when the customer *i* expects to be served

*P_e_*: Cost of waiting for the unit time if the vehicle arrives at customer node in advance (yuan/min)

*P_l_*: Cost of punishing for the unit time if the vehicle is late to the customer node (yuan/min)

: Time at which the *k^th^* vehicle arrives at point *i*

: Time at which the *k^th^* vehicle leaves point *i*

**Reviewer#1, Concern # 4:** Figure 4 is missing, please check Figure no.

**Author response:** Thank you for your constructive and helpful suggestion. I readjusted the diagram and normalized the number.

**Reviewer#1, Concern # 5:**  if Δw<0 is occurs which is realistic in the section 2.3, subsequent results will result in errors, Especially in section 2.4. Furthermore, do w in the section 2.3 and TL in the section 2.4 have the same meaning?

**Author response:** Thank you for your constructive and helpful suggestion. We have restandardized the units of and to $W_{O}\text{ and }W_{L}$in section 2.2, and we've defined the relationship between $W_{L}$and *w* in section 2.5.

*w*: Temperature of controlled temperatures (Celsius)

: Ambient thermodynamic temperature outside, thermodynamic temperature (K)

: Thermodynamic temperature of controlled temperatures *w* (K),

Because Δw<0 is occurs which is realistic, so we updated the food safety loss *F_ijw_* :

$$FS_{ijkw}\text{=}ds\cdot q_{j}\cdot\Delta W^{2}(T_{jk}^{leave}-T_{ik}^{leave}),\text{ }\Delta W=\left\{\begin{matrix} w-W_{\min},w\geq W_{\min} \\ 0,\text{ }w<W_{\min}\text{ } \end{matrix}\right.,i\in\mathbf{N},j\in\mathbf{N}'$$

If $w\geq W_{\min}$, it will lead to the growth of microorganisms in food and increase the cost of food safety loss; otherwise the loss of food safety remains the same with the absence of microbial growth, but the cost of temperature control will increase significantly with the decrease of temperature.

**Reviewer#1, Concern # 6:** Specify the relationship between COP, gw , and θw , and the Formula gw=gf*Tfw*θw in section 2.4 is wrong

**Author response:** Thank you for your constructive and helpful suggestion. We have identified the relationship between COP, $\theta_{w}$, $f_{kw}$ and $g_{kw}$ in section 2.5.

$COP\text{=}\frac{W_{\text{L}}}{W_{O}-W_{L}}$ (5);

$\theta_{w}$ at different temperature control *w* is obtained by *COP* ratio;

According to the method of Ref. [22,29], and to make the calculations more realistic, we set the benchmark: Assuming that the outside temperature is $30^{\circ}\text{C}$, and the cost of cold storage temperature to $10^{\circ}C$ is one unit, i.e.,$\theta_{w}=1\text{ }(w=10^{\circ}C)$ . In this case, the refrigeration equipment of refrigerated vehicle adopts independent units, and the unit time fuel consumption of refrigerated equipment under full load(2000kg) is 2.4(L/h) which is obtained through experimental simulation, and (L/kg.h). then the cooling cost coefficient of $9^{\circ}C$, $\theta_{w}$(w=9), is 18.87/17.63=1.07, and the cooling cost coefficients for other controlled temperatures can be calculated. The of different temperature control *w* can be calculated:

$f_{kw}=f_{k(w=10^{\circ}C)}*\theta_{w}=1.2*10^{-3}*\theta_{w}$ (6)

Table 1. The values of COP, , and at different temperatures

| ***w*** | ***COP*** |  |  | ***w*** | ***COP*** |  |  |
| --- | --- | --- | --- | --- | --- | --- | --- |
| 10 | 14.15 | 1.00 | 1.20E-03 | 2 | 11.96 | 1.58 | 1.58E-03 |
| 9 | 17.63 | 1.07 | 1.07E-03 | 1 | 11.42 | 1.65 | 1.65E-03 |
| 8 | 16.53 | 1.14 | 1.14E-03 | 0 | 10.92 | 1.73 | 1.73E-03 |
| 7 | 15.56 | 1.21 | 1.21E-03 | -1 | 10.46 | 1.80 | 1.80E-03 |
| 6 | 14.68 | 1.28 | 1.28E-03 | -2 | 10.04 | 1.88 | 1.88E-03 |
| 5 | 13.90 | 1.36 | 1.36E-03 | -3 | 9.64 | 1.96 | 1.96E-03 |
| 4 | 13.19 | 1.43 | 1.43E-03 | -4 | 9.28 | 2.03 | 2.03E-03 |
| 3 | 12.55 | 1.50 | 1.50E-03 | -5 | 8.93 | 2.11 | 2.11E-03 |

Table 1 lists the values of COP, , and $\theta_{w}$ at different temperatures *w* (). The cost of temperature control per unit time at different temperatures can be obtained by solving the equation: (7)

**Reviewer#2, Concern # 1:** In the section 2.2, the paper explains the relationship between the driving speed and the departure time. The travel time function is formulated. However, in the following section, the travel time function is useless because the paper partition the route into a sufficiently short section and the vehicle speed on this short section is set to be constant.

**Author response:** Thank you for your constructive and helpful suggestion. There are errors in the previous description, so I redescribe the change of speed under the time-varying network in section 2.3. I have modified the calculation method of driving time under time-varying network.

In a time-varying network, vehicle speeds vary with the time period. Therefore, travel time is difficult to calculate and needs to be rationally dealt with. Based on the previously proposed cross-time period travel time calculation methods [23,27,28], the real-time speed of a vehicle within a sufficiently short distance is used as its fixed speed within the time period. The following shows a step function for the speed () at various times on the road (*i, j*):

(1)

**Reviewer#2, Concern # 2:** In the section 2.3, $W_{\min}$ is the temperature at which no microorganisms grow (Celsius) but it is not the minimum value of controlled temperature *w*. if the condition $w<W_{min}$ occurs, the formula (3) will get the wrong result that the food safety loss increases with the decrease of *w*.

**Author response:** Thank you for your constructive and helpful suggestion. We updated the food safety loss *F_ijw_* :

$$FS_{ijkw}\text{=}ds\cdot q_{j}\cdot\Delta W^{2}(T_{jk}^{leave}-T_{ik}^{leave}),\text{ }\Delta W=\left\{\begin{matrix} w-W_{\min},w\geq W_{\min} \\ 0,\text{ }w<W_{\min}\text{ } \end{matrix}\right.,i\in\mathbf{N},j\in\mathbf{N}'$$

If $w\geq W_{\min}$, it will lead to the growth of microorganisms in food and increase the cost of food safety loss; otherwise the loss of food safety remains the same with the absence of microbial growth, but the cost of temperature control will increase significantly with the decrease of temperature.

**Reviewer#2, Concern # 3:** In the formula (4), is not right that leads to the wrong formula (4). The formula (5) also has the same mistake.

**Author response:** Thank you for your constructive and helpful suggestion. We updated the formulas:

$FS_{ijkw}\text{=}ds\cdot q_{j}\cdot\Delta W^{2}(T_{jk}^{leave}-T_{ik}^{leave}),\text{ }\Delta W=\left\{\begin{matrix} w-W_{\min},w\geq W_{\min} \\ 0,\text{ }w<W_{\min}\text{ } \end{matrix}\right.,i\in\mathbf{N},j\in\mathbf{N}'$ (3)

The driving time is calculated as:$T_{jk}^{leave}-T_{ik}^{leave}$;

Due to the differences in customer demand, the total safety loss not only relates to the controlled temperature and the distribution time but also depends on the customer service order.

The total safety loss cost of all vehicles during distribution can be expressed as:

(4)

**Reviewer#2, Concern # 4:** In the section 2.4, the units of and should be clear, Celsius or Kelvin (K).

**Author response:** We are very grateful to the reviewee for pointing out such subtle mistake and we corrected it in the new manuscript. We restandardized the units of and to $W_{O}\text{ and }W_{L}$in section 2.2, and explained the relationship between them in the section2.5

*w*: Temperature of controlled temperatures (Celsius)

: Ambient thermodynamic temperature outside, thermodynamic temperature (K)

: Thermodynamic temperature of controlled temperatures *w* (K),

**Reviewer#2, Concern # 5:** In table 1, the value of COP is not right if.

**Author response:** We are very grateful to the reviewee for pointing out such subtle mistake and we corrected it in the new manuscript, and Table 1 has been modified in section 2.5.

If the outside thermodynamic temperature $W_{O}$ is 303 K(outside temperature $\text{temp=30}^{\circ}\text{C}$) ,and the target controlled thermodynamic temperature $W_{L}$is 283K($w=10^{\circ}C$), the values of COP is $COP=\frac{283}{303-283}=14.15$.

Table 1. The values of COP, , and at different temperatures

| ***w*** | ***COP*** |  |  | ***w*** | ***COP*** |  |  |
| --- | --- | --- | --- | --- | --- | --- | --- |
| 10 | 14.15 | 1.00 | 1.20E-03 | 2 | 11.96 | 1.58 | 1.58E-03 |
| 9 | 17.63 | 1.07 | 1.07E-03 | 1 | 11.42 | 1.65 | 1.65E-03 |
| 8 | 16.53 | 1.14 | 1.14E-03 | 0 | 10.92 | 1.73 | 1.73E-03 |
| 7 | 15.56 | 1.21 | 1.21E-03 | -1 | 10.46 | 1.80 | 1.80E-03 |
| 6 | 14.68 | 1.28 | 1.28E-03 | -2 | 10.04 | 1.88 | 1.88E-03 |
| 5 | 13.90 | 1.36 | 1.36E-03 | -3 | 9.64 | 1.96 | 1.96E-03 |
| 4 | 13.19 | 1.43 | 1.43E-03 | -4 | 9.28 | 2.03 | 2.03E-03 |
| 3 | 12.55 | 1.50 | 1.50E-03 | -5 | 8.93 | 2.11 | 2.11E-03 |

**Reviewer#2, Concern # 6:** Why assume if and?

**Author response:** Thank you for your constructive and helpful suggestion.

We mainly refer to literatures[22,29] to find the benchmark of temperature control cost——When the outside thermodynamic temperature $W_{O}$ is 303 K(outside temperature $\text{temp=30}^{\circ}\text{C}$) ,and the target controlled thermodynamic temperature $W_{L}$is 283K($w=10^{\circ}C$), the temperature control cost is the standard, and the cooling cost coefficient in the control temperature of *w* $\theta_{w}=1$. At this case, we can we estimate that the unit time fuel consumption of refrigerated equipment under full load(2000kg) is 2.4(L/h) which is obtained by looking up relevant data on the Internet, and we can get the fuel consumption of unit delivery time and unit fresh product where the *k^th^* vehicle travels in the control temperature of $w=10^{\circ}C$:$f_{kw}=1.2\times10^{-3}$(L/kg.h). According to this standard, the cost under other temperature control can be calculated. Table 1 lists the values of COP, $f_{kw}$, and $\theta_{w}$ at different temperatures *w* ($^{\circ}\text{C}$).

[22] Wang Y, Zhang J. Optimization Method Study of Fresh Good Logistics Distribution Based on Time Window and Temperature Control . Control and Decision [Online]. Available: DOI: 10.13195/j.kzyjc.2018.1662

[29] Wang J, Liu H T, Zhao R. The optimization of cold chain operation based on fresh food safety[J]. Systems Engineering-Theory & Practice, 2018, 1:122-134

**Reviewer#2, Concern # 7:** In formula (5), why is the linear relationship between the customer demand and temperature control cost? As we know, the customer demand actually affects the temperature control cost. But the relationship is absolutely not linear. For example, the temperature control cost for transporting one hundred commodities is absolutely not 100 times that for transporting one commodity.

**Author response:** Thank you for your constructive and helpful suggestion. We mainly refer to the calculation method of temperature control cost in literature [16]. For the convenience of calculation, there is a linear relationship between temperature control cost and weight of demand. Under the condition of fixed temperature control, the larger the weight of refrigerated fresh products, the higher the fuel consumption required for temperature control and the higher the cost. Literature [16] explains the operation steps in detail. I also modified it accordingly in section 2.5:

According to formula (5), COP at different temperature-controlled temperatures w can be calculated. According to the method of Ref. [22,29], and to make the calculations more realistic, we set the benchmark: Assuming that the outside temperature is 25, and the cost of cooling to 10 is one unit, i.e., =1 (w=10). In this case, the refrigeration equipment of refrigerated vehicle adopts independent units, and the unit time fuel consumption of refrigerated equipment under full load is 2.4(L/h) which is obtained through experimental simulation, and (L/kg.h). then the cooling cost coefficient of 9, (w=9), is 18.87/17.63=1.07, and the cooling cost coefficients for other controlled temperatures can be calculated. The of different temperature control *w* can be calculated:

$f_{kw}=f_{k(w=10^{\circ}C)}*\theta_{w}=1.2*10^{-3}*\theta_{w}$ (6)

Table 1 lists the values of COP, , and at different temperatures w (). The cost of temperature control per unit time at different temperatures can be obtained by solving the equation:

(7)

As you have described, the linear relationship between customer demand and temperature control cost may not be in line with the reality. To simulate their real relationship is also my research direction in the future, and I put this work in the conclusion part.

[22] Wang Y, Zhang J. Optimization Method Study of Fresh Good Logistics Distribution Based on Time Window and Temperature Control. Control and Decision [Online]. Available: DOI: 10.13195/j.kzyjc.2018.1662

**Reviewer#2, Concern # 8:** In formula (8) and (9), the product of should be equal to. Why not use instead of to simplify the formula (8) and (9).

**Author response:** Thank you for your constructive and helpful suggestion. Not all road lengths are integer multiples of. We design a route partitioning method in section 2.3: The route (*i, j*) is partitioned to a number of $H=\left\lceil d_{ij}\text{/}\vartheta\right\rceil$route sections ( means rounding up to the nearest integer) according to $\vartheta$ . The first H-1 route sections have a length of $\vartheta$, while the last section has a length of $d_{ij}\text{-}\vartheta\text{*（}H\text{-1）}$. Because not all the route sections length are $\vartheta$, the last section might be less than $\vartheta$, the paper doesn’t use instead of to simplify the formula (8) and (9).

Since all road lengths are not equal, we use to represent the length of route section. At the same, due to the $v_{ijk}^{R}$ and $t_{ijk}^{R}$ of each route section is different, so the carbon emission rate and carbon emission of each route section are correspondingly different, and it is reasonable that use .

**Reviewer#2, Concern # 9:** In the section 2.7, what is the meaning of in formula (10)?

**Author response:** Thank you for your constructive and helpful suggestion. is the cost of safety loss. Further explanation: The total loss cost is the product of the unit price of goods and the amount of loss. To illustrate further, we have modified the total cost of the security loss FS in section 2.4:

(4)

I also have modified the target function accordingly (14):

(14)

**Reviewer#2, Concern # 10:** Formula (12) is wrong. It cannot ensure that the vehicle arrives at and leaves from the same node.

**Author response:** Thank you for your constructive and helpful suggestion. I have modified the formula (12) to formula (16):

（16）

Formula (16) indicates that the vehicle arrives at and leaves from the same node.

**Reviewer#2, Concern # 11:** Formula (18) and (19) cannot reflect the time window constraints properly. For example, if, the waiting time still exists that means the vehicle has to wait for a while even it arrives within the time window.

**Author response:** Thank you for your constructive and helpful suggestion. I updated the method the customer point window constraint in section 2.1: There is also a service time-window requirement. A vehicle can reach a client point to provide service at an earlier or later time but then will pay a certain penalty.

I have solved the customer point time window constraint by increasing the penalty cost in section 2.7:

**2.7 Vehicle penalty cost**

This penalty cost is incurred by the vehicle as a result of violating the client’s time window during the distribution process. Client *i* accepts service outside the time window, but there is a penalty cost for failing to provide service within the appointed time, and the total penalty cost *PC* equals to

(13)

: Time window when the customer *i* expects to be served

*P_e_*: Cost of waiting for the unit time if the vehicle arrives at customer node in advance (yuan/min)

*P_l_*: Cost of punishing for the unit time if the vehicle is late to the customer node (yuan/min)

: Time at which the *k^th^* vehicle arrives at point *i*

: Time at which the *k^th^* vehicle leaves point *i*

**Reviewer#2, Concern # 12:** In the proposed model, it lacks the constraints for and. It means that if, must be 1. Whereas, if, can be 1 or 0.

**Author response:** Thank you for your constructive and helpful suggestion. I have modified the constraints for and in section 2.8:

（22）

（23）

**Reviewer#2, Concern # 13:** In the proposed model, constraints that vehicles must return the distribution center are omitted.

**Author response:** Thank you for your constructive and helpful suggestion. I have modified it as required:

（24）

Eqn. (24) shows that all vehicles start and end at the distribution center.

**Reviewer#2, Concern # 14:** In section 4.2.2, there is no figure 4.

**Author response:** Thank you for your constructive and helpful suggestion. I readjusted the diagram and normalized the number.

**Reviewer#2, Concern # 15:** The discussion for figure 2 is not appropriate because there is no temperature coordinate in figure 2.

**Author response:** Thank you for your constructive and helpful suggestion. I modified Figure 2 to Figure 3, and added a temperature annotation in Figure 3 to illustrate.

**Reviewer#2, Concern # 16:** Figure 5 should be Figure 4.

**Author response:** Thank you for your constructive and helpful suggestion. There is some error in figure numbering in the article, so I have modified the number of the Figure as required.

**Reviewer#2, Concern # 17:** $5^{\circ}C$is the optimal refrigerated temperature observed in figure 3. Why not set the controlled refrigerated temperature to in the section 4.2.3.

**Author response:** Thank you for your constructive and helpful suggestion. I have modified the number of the Figure as required. After modification, the optimal temperature control temperature is $1^{\circ}C(w=1^{\circ}C)$, so the following experiments in this paper are calculated according to this case($w=1^{\circ}C$).

**Reviewer#2, Concern # 18:** In this paper, several variables are not defined before they are used (e.g.,,,,, and etc.) and some variable definitions are duplicated (e.g., and, and). A lot of variables are higher than the text and not italic. The subscripts of some variable are wrong (e.g., ). The authors should be checked all paper carefully and correct all mistakes

**Author response:** Thank you for your constructive and helpful suggestion. We updated the manuscript by declaring all variables in section 2.2, at the same time, the duplicated variables are removed such as TL, WL, w and so on. is the length of the route section, H is the route section number, and They have different meanings.

Matrices are bolded and variables are italicized. We have unified the variable *W_L_* instead of *T_L_*, and We corrected the variable in section 2.4:

$FS=P\cdot ds\cdot\Delta W^{2}\sum_{k=1}^{K} \sum_{j=1}^{n} q_{j}(T_{jk}^{leave}-T_{0k}^{leave})\text{ }\Delta W=\left\{\begin{matrix} w-W_{\min},w\geq W_{\min} \\ 0,\text{ }w<W_{\min}\text{ } \end{matrix}\right.,i\in\mathbf{N},j\in\mathbf{N}'$ (4)

I have rearrangement and corrected all the variables: Matrices are bolded, and variables are italicized.
